# Case report: Catastrophic event: neonatal gastric perforation and complication of capillary leak syndrome

**DOI:** 10.3389/fped.2023.1257491

**Published:** 2023-09-20

**Authors:** Jie Li, Hongping Lu, LinJun Yu, Haiting Li, Xiyang Chen, Caie Chen, Enfu Tao

**Affiliations:** ^1^ Department of Neonatology and NICU, Wenling Maternal and Child Health Care Hospital, Wenling, China; ^2^ Department of Neonatology, Taizhou Hospital of Zhejiang Province, Wenzhou Medical College, Linhai, China; ^3^ Department of Pediatric Surgery, Taizhou Hospital of Zhejiang Province, Wenzhou Medical College, Linhai, China; ^4^ Department of Science and Education, Wenling Maternal and Child Health Care Hospital, Wenling, China

**Keywords:** neonatal gastric perforation, capillary leakage syndrome, sepsis, septic shock, hyperpermeability

## Abstract

Neonatal gastric perforation (NGP) is a rare, but life-threatening condition that can lead to serious conditions, such as capillary leak syndrome (CLS). Here, we present the case of a preterm male infant with NGP complicated by CLS after stomach repair. The patient was born at 33 2/7 weeks, weighed 1,770 g, and was diagnosed with respiratory distress syndrome. On the fourth day of life, the patient presented with distention and an unstable cardiovascular system. Routine blood tests revealed a white blood cell count of 2.4 × 10^9^/L. Chest and abdominal radiography revealed a pneumoperitoneum, suggesting a gastrointestinal perforation. The patient was urgently transferred to a tertiary hospital for exploratory laparotomy, where a 2 cm diameter perforation was discovered in the stomach wall and subsequently repaired. Pathological findings indicated the absence of a muscular layer in the stomach wall. The patient unexpectedly developed CLS postoperatively, leading to multiorgan dysfunction and eventual death. The underlying pathological mechanism of NGP-induced CLS may be related to severe chemical peritonitis, sepsis, endothelial glycocalyx dysfunction, enhanced systemic inflammation, and translocation of the gut microbiota, causing endothelial hyperpermeability. Notablely, abdominal surgery itself can be a significant triggering factor for CLS occurrence. Complications of NGP and CLS are extremely dangerous. Investigating the mechanism by which NGP triggers CLS could potentially improve the prognosis. Conservative treatment for pneumoperitoneum secondary to gastric perforation may be a reasonable option, especially when the condition of the patient is unstable.

## Introduction

Neonatal gastric perforation (NGP) is a rare but life-threatening condition (1). The etiologies of NGP remain controversial and elusive; however, the absence of gastric musculature is one of the major causes of NGP (2). The causes of death for NGP are multiple, and infections are the most common cause (2). In addition, hypercytokinemia caused by sepsis or septic shock may lead to a more complicated and severe condition called capillary leak syndrome (CLS) (3) that is characterized by hypoalbuminemia, hypotension, generalized edema, and acute kidney ischemia caused by the leakage of fluids and macromolecules into tissues due to the increased permeability of capillary endothelial cells (3, 4). However, to date, NGP-induced CLS has not been reported in the literature.

Herein, we described the case of a preterm infant with NGP due to the absence of gastric wall musculature who developed CLS after stomach repair.

## Case presentation

**Figure 1:**
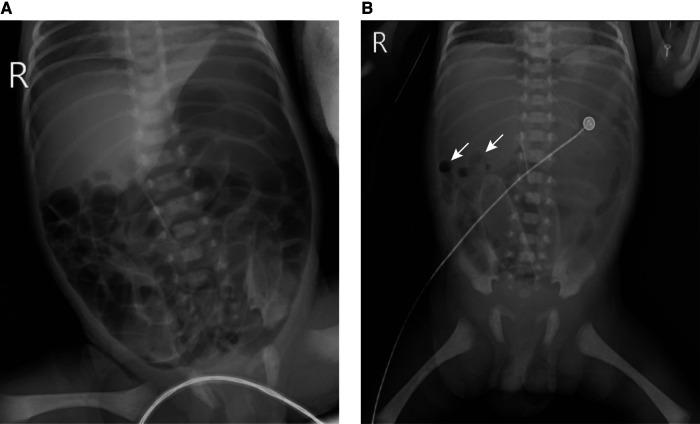
Abdominal x-ray findings of the neonatal gastric perforation before and after perforation. (**A**) The abdominal x-ray findings at postnatal day 4 AM before perforation; (**B**) the abdominal x-ray findings after perforation. The white arrow indicates pneumoperitoneum.

**Figure 2:**
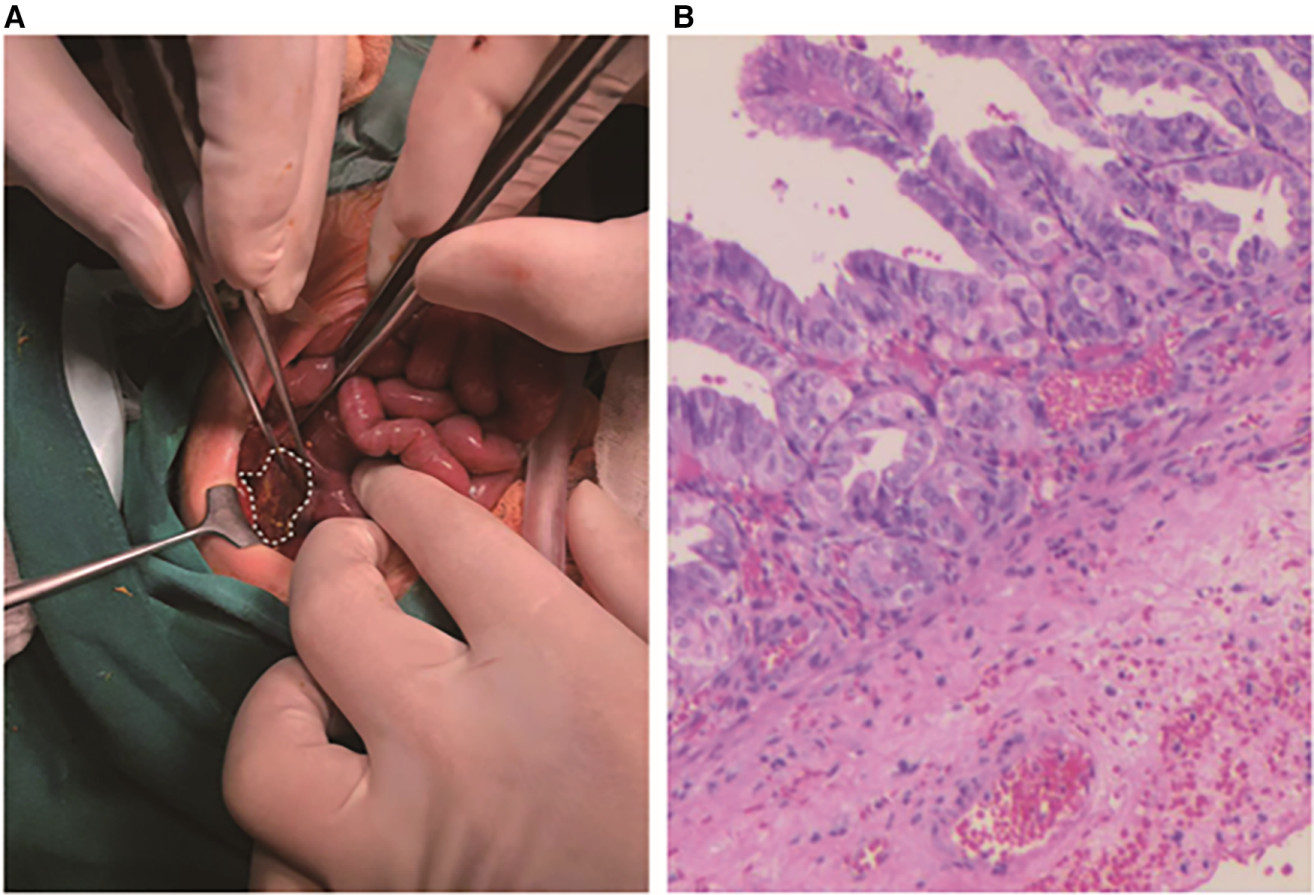
Intraoperative findings and pathological report of neonatal gastric perforation. (**A**) Intraoperative findings of neonatal gastric perforation. (**B**) Pathological report of neonatal gastric perforation. Microscopic examination revealed gastric mucosal tissue around the perforation site without any muscle tissue. The perforation site is indicated by the white dashed line.

A male preterm infant with a gestational age of 33 2/7 weeks and birth weight of 1,770 g was delivered by cesarean section because of severe maternal preeclampsia. The patient was dignosed with respiratory distress syndrome. On postnatal day (PND) 2, the patient presented with multiple episodes of apnea and was supported by nasal intermittent positive pressure ventilation (NIPPV). In addition, a slight abdominal distension was observed. On PND 3, the gastrointestinal decompression tube showed coffee ground retention accompanied by abdominal distension. On the morning of PND 4, respiratory support was alternated to humidified and heated high-flow nasal cannula. However, the gastrointestinal decompression tube retained drainage of the coffee grounds, accompanied by significant abdominal distension and diminished bowel sounds. Chest and abdominal radiography revealed pneumatosis in the gastrointestinal tract (Figure 1A). At noon, the patient suddenly presented with cardiovascular system instability, with a pulse rate of 180–190 beats/min, blood pressure of 63/36 mmHg, skin mottling, and capillary refill time of 5 s. The patient rapidly developed sepsis, septic shock, leukopenia (white blood cell count of 2.4 × 10^9^/L), hypoxemia (PO_2_ of 50.4 mmHg), metabolic acidosis (pH value of 7.32, base excess −6.20 mmol/L), hyperlactacidemia (lactic acid level of 5.2 mmol/L), hypokalemia (potassium level of 3.16 mmol/L) and hyperglycemia (blood glucose level of 8 mmol/L). Emergency medical treatments were implemented immediately. However, the condition was not ameliorated, and lactic acid levels were persistently elevated. Chest and abdominal radiograph revealed pneumoperitoneum, indicating gastrointestinal perforation (Figure 1B). The patient was transferred to a tertiary hospital, and emergency exploratory laparotomy was immediately performed. A perforation of approximately 2 cm was found on the anterior wall of the stomach (Figure 2A) and pathological report revealed gastric mucosal tissue around the perforation site without any muscle tissue (Figure 2B). Surgical intervention involved stomach repair, appendix removal, and abdominal irrigation. No surgical complications occurred. However, the blood pressure of the patient was unstable postoperatively, with oliguria and an increased heart rate, indicating hypovolemia. Despite multiple attempts at volume expansion and treatment with vasoactive drugs, no improvement was observed. In addition, the patient presented with electrolyte imbalances, as well as edema and hypoproteinemia (serum albumin level: 15.1 g/L), indicating CLS. The patient received treatment involving the administration of albumin, plasma, hydroxyethyl starch, correction of electrolyte imbalances, and adrenaline to elevate blood pressure. Unfortunately, the condition worsened with the development of anuria, widespread edema, and decreased blood oxygen saturation. Despite efforts to correct this situation, the patient ultimately experienced multiple organ failure. The family eventually decided to discontinue the treatment, and the patient passed away. The representative laboratory examination results before perforation, post-perforation, and after surgery for gastric perforation are summarized in Table 1.

**Table 1:** Representative laboratory examination results pre-perforation, post- perforation, and post-surgery for gastric perforation.

Indicators	Pre-perforation	Post- perforation	Post-surgery
Complete blood count			
White blood cell (×10^9^/L)	4.9	2.0	5.2
Hemoglobin (g/L)	177	122	101
Neutrophil (%)	26	67.7	71.7
Lymphocyte (%)	65.7	26.6	14.9
Platelets (×10^9^/L)	168	37	36
High-sensitivity C-reactive protein (mg/L)	<0.5	85.6	82.9
Arterial blood gas analysis			
pH	7.32	7.41	6.72
PaCO_2_ (mmHg)	38.9	32	92
PaO_2_ (mmHg)	51.1	52	20
Bicarbonate (mmol/L)	19.4	19.6	11.7
Base excess (mmol/L)	−6.2	−5.1	−22.7
Lactic acid (mmol/L)	5.2	8.53	13.65
Blood glucose (mmol/L)	8.0	3.9	9.0
Electrolytes			
Calcium (mmol/L)	1.04	0.98	1.16
Sodium (mmol/L)	134.7	129.9	124.2
Potassium (mmol/L)	3.16	3.54	4.81
Blood biochemistry			
Alanine aminotransferase (IU/L)	5	N/A	7
Aspartate aminotransferase (IU/L)	45	N/A	52
Albumin (g/L)	31	N/A	17.5
Creatinine (μmol/L)	65	91	84
Blood urea nitrogen (mmol/L)	5	7.66	5.7
Blood culture	Negative	N/A	Negative
Procalcitonin (ng/ml)	0.35	246.80	N/A
Coagulation function			
Prothrombin time (s)	35.5	22.9	30.6
International normalized ratio	3.0	2.07	2.99
Activated partial thromboplastin time (s)	43.2	48	74.6
Thrombin time (s)	17	16.4	16.1
Fibrinogen (g/L)	1.46	3.19	1.86

PaCO_2_, partial pressure of carbon dioxide in arterial blood; PaO_2_, partial pressure of oxygen in arterial blood.

## Discussion

We present a rare case of NGP caused by the absence of the stomach muscle layer. Unfortunately, the patient, compilicated by CLS after gastric repair, died due to multiple organ failure. This is the first documented case of NGP leading to the development of CLS in the literature. Although this was a fatal case, several urgent issues need to be addressed.

The pathological mechanisms underlying the development of CLS from NGP are not fully understood. After gastric perforation, the release of bacteria, stomach acid, digestive enzymes, and partially digested milk into the peritoneal cavity can cause severe chemical peritonitis, which can rapidly progress to bacterial peritonitis and sepsis if left untreated (5, 6). Sepsis is an evolving process; the systemic inflammatory response to infection is usually associated with hypoperfusion, followed by tissue injury and organ failure (7). The endothelium covering the surfaces of blood vessels and organs is one of the most damaged organs during sepsis (8). Endothelial glycocalyx (eGC) is a carbohydrate-rich layer lining the vascular endothelium, playing an important role in vascular barrier function and cell adhesion properties, serving also as a mechano-sensor for blood flow (8). Shedding or degradation of the eGC is considered a crucial pathological process that leads to microvascular dysfunction (8, 9). Destruction of eGC increases macromolecule permeability and leukocyte adhesion in the subcutaneous microcirculation (10) and may contribute to alterations in microvascular rheology; affecting capillary blood flow and ultimately tissue perfusion (11). Furthermore, several clinical signs, such as uncontrolled clotting activation, thrombosis, edema, local hypoxia, and ischemia, are initiated after glycocalyx degradation in sepsis (12). In addition, after gastric perforation, the infection caused by pathogenic microorganisms can trigger endothelial cell activation, possibly damaging its structure and function (13). Moreover, translocation of microbial molecules from the gut into the systemic circulation leads to enhanced systemic inflammation (14). Ultimately, the vascular barrier is impaired (9). Endothelial hyperpermeability causes interstitial edema, raising interstitial pressure and worsening tissue hypoperfusion; this can result in organ injury and life-threatening organ failure (9). More importantly, vascular endothelium hypermeability can lead to CLS, a rare and severe condition. Notablely, CLS developed after gastric repair in this case. Reports indicate that 20% of very low birth weight infants with necrotizing enterocolitis (NEC) develop CLS following surgical treatment (15), suggesting that abdominal surgery itself can be a significant triggering factor for CLS occurrence.

Prompt surgical laparotomy is encouraged and can be lifesaving (2). In the context of NGP, repairing a ruptured stomach and washing out the abdominal cavity is of great importance (16). However, the patient developed severe CLS after gastric repair and eventually died, raising the question of the appropriate timing of surgery. Historically, pneumoperitoneum has been considered an absolute indication for laparotomy. However, Upadhyaya et al. found that pneumoperitoneum is not an absolute indication for exploratory laparotomy in NEC cases (17). In a large cohort of patients with NGP, of the 66 patients who underwent surgical laparotomy, 26 (39.4%) died (2), suggesting the potential surgical risk for mortality. Conservative management of neonatal pneumoperitoneum with a peritoneal drain has been proposed in situations where the patient is unstable or fragile or shock has not been corrected (18). Clinical evidence showed that intestinal perforation can be successfully treated conservatively (19). Moreover, if the hemodynamics are stable, even in cases of rupture of the entire stomach and duodenum, pneumoperitoneum could be successfully treated through exploratory laparotomy (20). Therefore, there may be a need to reexamine the indications for surgery in cases of digestive perforation with pneumoperitoneum. Conservative treatment for pneumoperitoneum secondary to gastric perforation may be a reasonable option, especially when the condition of the patient is unstable.

## Conclusion

In conclusion, NGP complicated by CLS is a rare and fatal condition. Emergency laparotomy may not be the best option for NGP when the condition of the patient is unstable.

## Data Availability

The original contributions presented in the study are included in the article/Supplementary Material, further inquiries can be directed to the corresponding author.
